# Serum SCFA and Nesfatin-1 Patterns in Inflammatory Bowel Disease: A Pilot Exploratory Study

**DOI:** 10.3390/jcm15072581

**Published:** 2026-03-27

**Authors:** Paul Grama, Tamás Ilyés, Naomi-Adina Ciurea, Radu-Alexandru Fărcaș, Simona Bățagă

**Affiliations:** 1 M3 Department, Discipline of Internal Medicine 1, George Emil Palade University of Medicine, Pharmacy, Science, and Technology of Târgu Mureș, 540139 Târgu Mureș, Romania; 2 Department of Gastroenterology, Târgu Mures Emergency Clinical County Hospital, 540136 Târgu Mureș, Romania; 3 Doctoral School, George Emil Palade University of Medicine, Pharmacy, Science, and Technology of Târgu Mureș, 540139 Târgu Mureș, Romania; 4 Department of Medical Biochemistry, “Iuliu Hațieganu” University of Medicine and Pharmacy, 400347 Cluj-Napoca, Romania; 5 2nd Department of Internal Medicine, “Iuliu Haţieganu” University of Medicine and Pharmacy, 400347 Cluj-Napoca, Romania

**Keywords:** inflammatory bowel disease, Crohn’s disease, ulcerative colitis, short-chain fatty acids, nesfatin-1, gut–brain axis, quality of life

## Abstract

**Background**: Short-chain fatty acids (SCFAs) support mucosal integrity and reduce inflammation, while nesfatin-1 is a neuropeptide with antiapoptotic, anti-inflammatory, antioxidant, and anorexigenic actions. Their roles in inflammatory bowel disease (IBD) and links to quality of life (QoL) are unclear. **Methods**: We conducted a cross-sectional study including adults with Crohn’s disease (CD), ulcerative colitis (UC), and healthy controls (HC). Serum total short-chain fatty acids and nesfatin-1 were measured using enzyme-linked immunosorbent assays (ELISA). Quality of life was assessed using the Inflammatory Bowel Disease Questionnaire (IBDQ). Group comparisons and correlation analyses were performed using non-parametric statistical methods. **Results**: Serum total SCFA concentrations did not differ significantly between patients with CD, UC, and HC (*p* = 0.29). Nesfatin-1 levels showed between-group variability, with lower values in CD compared with healthy controls, while patients with UC showed intermediate and variable levels (*p* = 0.064). An inverse correlation between SCFAs and nesfatin-1 was observed in UC and in the combined IBD cohort, but not in CD. Quality of life was comparably impaired in CD and UC. No statistically significant associations were observed between serum SCFAs or nesfatin-1 and IBDQ scores. **Conclusions**: In this pilot exploratory study, circulating SCFAs and nesfatin-1 showed distinct patterns across IBD subtypes, with evidence of subtype-specific associations between these biomarkers. However, no relationship with quality of life was demonstrated. Larger longitudinal studies are required to confirm these findings and clarify their clinical relevance.

## 1. Introduction

Inflammatory bowel diseases (IBD), comprising Crohn’s disease (CD) and ulcerative colitis (UC), are chronic relapsing inflammatory conditions of the gastrointestinal tract. IBD pathogenesis involves complex interactions between the gut microbiota, the intestinal immune system, and host regulatory molecules. Non-invasive diagnostic markers play a central role in assessing intestinal inflammation in IBD. Current European Crohn’s and Colitis Organisation’s (ECCO) recommendations support the use of fecal calprotectin as a first-line tool to detect mucosal inflammation and to monitor disease activity. Serum CRP and other inflammatory markers also assist clinical decision-making [1].

Short-chain fatty acids (SCFAs), primarily acetate, propionate, and butyrate are produced by colonic bacterial fermentation of dietary fiber [2]. In IBD, dysbiosis may lead to reduced SCFA production by lowering butyrate-producing bacteria. This phenomenon may correlate to disease severity, on account of the SCFAs being responsible for the exertion of anti-inflammatory and epithelial barrier-protective effects, and of their central role in immune regulation in IBD [3,4].

Nesfatin-1, a peptide obtained from the precursor nucleobindin-2 (NUCB2), is a relatively newly identified peptide [5]. Originally detected in the hypothalamus as a potent anorexigenic neuropeptide, it has also been found to exert anti-inflammatory, antioxidant and antiapoptotic effects [6]. In addition to the central nervous system, nesfatin-1 is also present in peripheral tissues, including the gastrointestinal tract, specifically in the gastric mucosal endocrine cells and in the enteric nervous system [7]. It helps with reducing food intake and is co-expressed in peripheral tissues with metabolic hormones (e.g., it is found in gastric X/A-like cells alongside ghrelin) [8]. This widespread distribution suggests that nesfatin-1 may serve as a point of convergence between the gut and the brain, affecting energy intake, metabolism and reactions to stress [9,10,11]. Previous studies suggest that circulating nesfatin-1 levels can change in pathological conditions. For example, nesfatin-1 is elevated in some cancers (including colorectal cancer, CRC), and has been associated with anorexia and cachexia in malignancy. Low leptin and nesfatin-1 levels were observed in some IBD cohorts, indicating a potential relation with malnutrition or disease chronicity [12].

Both SCFAs and nesfatin-1 play significant roles in gut–brain axis signaling [13]. SCFAs are known for their ability to activate G-protein coupled receptors (FFAR2/GPR43, FFAR3/GPR41) [14] on intestinal enteroendocrine cells. This stimulates the release of peptides such as glucagon-like peptide-1 (GLP-1) and peptide YY [15], which, in turn, influence appetite and immune function [16].

This study explores the potential associations between SCFAs and nesfatin-1 in IBD subtypes without prespecifying a directional relationship given the limited and heterogeneous data available in the literature. Although SCFAs and various gut hormones have been studied in patients with IBD (e.g., SCFAs with leptin, GLP-1 and nesfatin-1 in patients with IBD and colon cancer) [12], previous work included neither a separate analysis of CD and UC nor patient-reported outcomes. The aim of this pilot exploratory study was to characterize serum total SCFA and nesfatin-1 profiles in patients with CD, patients with UC, and healthy controls (HC). The potential associations between these biomarkers and patient-reported quality of life were further explored, once again without prespecifying directional effects.

## 2. Materials and Methods

### 2.1. Study Design and Participants

A cross-sectional observational study was conducted on adult patients with IBD (CD and UC) and healthy controls. All patients with IBD were recruited from the Gastroenterology Department of Târgu Mureș Emergency Clinical County Hospital (Târgu Mureș, Romania) during routine clinic visits. Patients were recruited consecutively between March 2024 and July 2024. All eligible patients attending the clinic during this period were invited to participate and no exclusion occurred beyond the predefined criteria. Diagnoses of CD or UC were established based on standard clinical, endoscopic and histopathological criteria. Exclusion criteria for the IBD groups included current use of antibiotics or probiotic supplements, recent surgery, pregnancy, or any concomitant inflammatory or neoplastic condition aside from IBD. Healthy controls were volunteers, without known gastrointestinal diseases, matched roughly to the patient groups in age and sex distribution. All participants provided informed consent, and the study protocol was approved by the Ethics Committee of our hospital. The reporting of this study followed the STROBE (Strengthening the Reporting of Observational Studies in Epidemiology) guidelines.

### 2.2. Biomarker Measurements and Data Collection

Fasting blood samples were collected in the morning (after an overnight fast of at least 10 h) in order to measure serum SCFA and nesfatin-1. Samples were collected into additive-free test tubes, centrifuged at 3000× *g* for 10 min, and the resulting serum was aliquoted and frozen at −80 °C until analysis. Serum nesfatin-1 and SCFAs were measured in batch using commercial enzyme linked immunosorbent assay (ELISA) kits (Human NES1 Nesfatin-1 ELISA Kit—Elabscience^®^, Wuhan Elabscience Biotechnology Co., Ltd., Wuhan, China, and SCFA Short-Chain Fatty Acids ELISA Kit—FineTest^®^, Wuhan FineTest Biotech Co., Ltd., Wuhan, China), with processing carried out according to the manufacturers’ instructions. The ELISA plates were read using an automated microplate reader (TECAN Sunrise™, Tecan Trading AG, Männedorf, Switzerland).

For the purpose of this study, SCFAs were measured in serum rather than fecal samples. Serum SCFAs were selected to reflect systemic exposure to microbial metabolites and their potential interaction with host metabolic and neuroendocrine pathways. Fecal SCFA analysis, while more directly reflecting luminal microbial activity, was not performed due to logistical constraints related to standardized stool collection and processing within the clinical setting. The assay used in this study quantified total SCFA concentrations and did not differentiate between individual SCFA components such as acetate, propionate, and butyrate.

Fecal calprotectin measurements were available only for a subset of patients with IBD and were not available for healthy controls. Therefore, calprotectin analyses were performed only as exploratory, descriptive assessments and were not intended for inferential comparisons. Stool samples were collected within seven days of the blood draw.

Disease activity was assessed using CDAI for CD and the Mayo score for UC.

As a measure of patient-reported outcome, we administered the validated Romanian-language version of the Inflammatory Bowel Disease Questionnaire (IBDQ) to patients with IBD, a validated instrument assessing disease-specific quality of life, yielding a total IBDQ score (higher scores indicating better quality of life). For this study, we analyzed the total IBDQ score, as our primary objective was to evaluate the overall quality of life. Use of the Inflammatory Bowel Disease Questionnaire, authored by Dr. Jan Irvine et. al., was made under license from McMaster University, Hamilton, Canada [17].

### 2.3. Statistical Analysis

Statistical analysis was carried out using RStudio Desktop (RStudio©, PBC v1.4.1106, Boston, MA, USA). Normality was assessed for all quantitative variables using the Shapiro–Wilk test, normally distributed data were analyzed using parametric tests, while non-parametric equivalents were used for non-Gaussian data. Group comparisons were carried out using the Kruskal–Wallis test or the Mann–Whitney U test, with Dunn’s test for post hoc comparisons where applicable. Associations between continuous variables were assessed using Spearman’s rank correlation coefficient. A value of *p* < 0.05 was considered significant for all analyses.

This study was designed as a pilot exploratory analysis; hence no prior sample size nor power calculation was performed. The sample size was pragmatic and based on available eligible participants during the study period. Given the exploratory nature of the study, no formal correction for multiple testing was prespecified. Instead, *p*-values are reported as obtained, and all results should be interpreted accordingly.

### 2.4. Data Visualization

**Figure 1:**
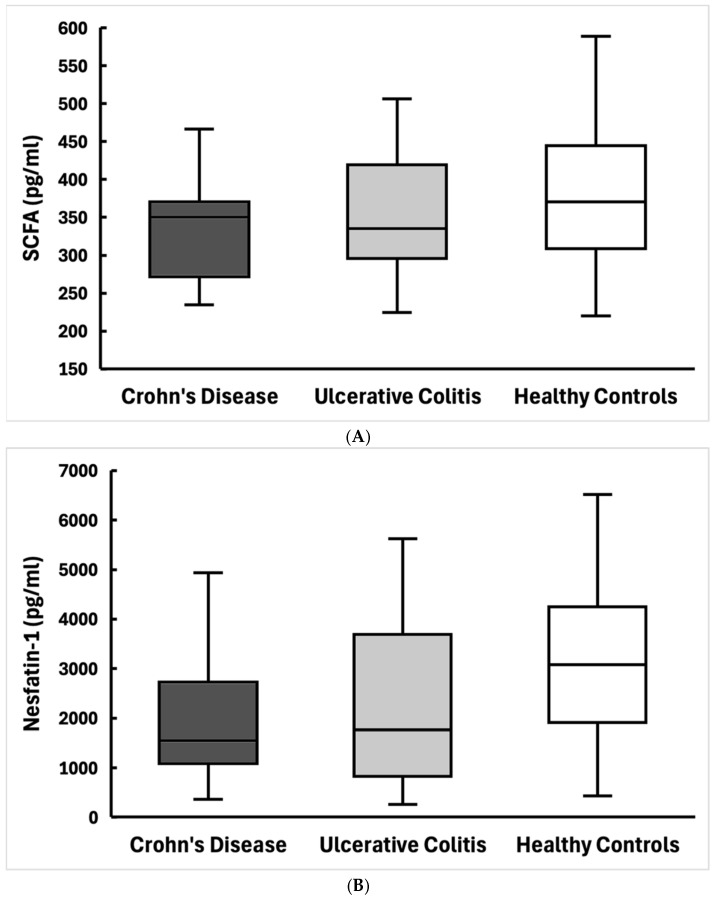
(**A**) Distribution of serum total short-chain fatty acid (SCFA) concentrations in patients with Crohn’s Disease (CD), Ulcerative Colitis (UC), and Healthy Controls (HC). Box-and-whisker plots show medians, interquartile ranges, and full ranges. (**B**) Distribution of serum nesfatin-1 levels in patients with Crohn’s disease (CD), ulcerative colitis (UC), and healthy controls (HC). Box-and-whisker plots display medians, interquartile ranges, and full ranges.

**Figure 2:**
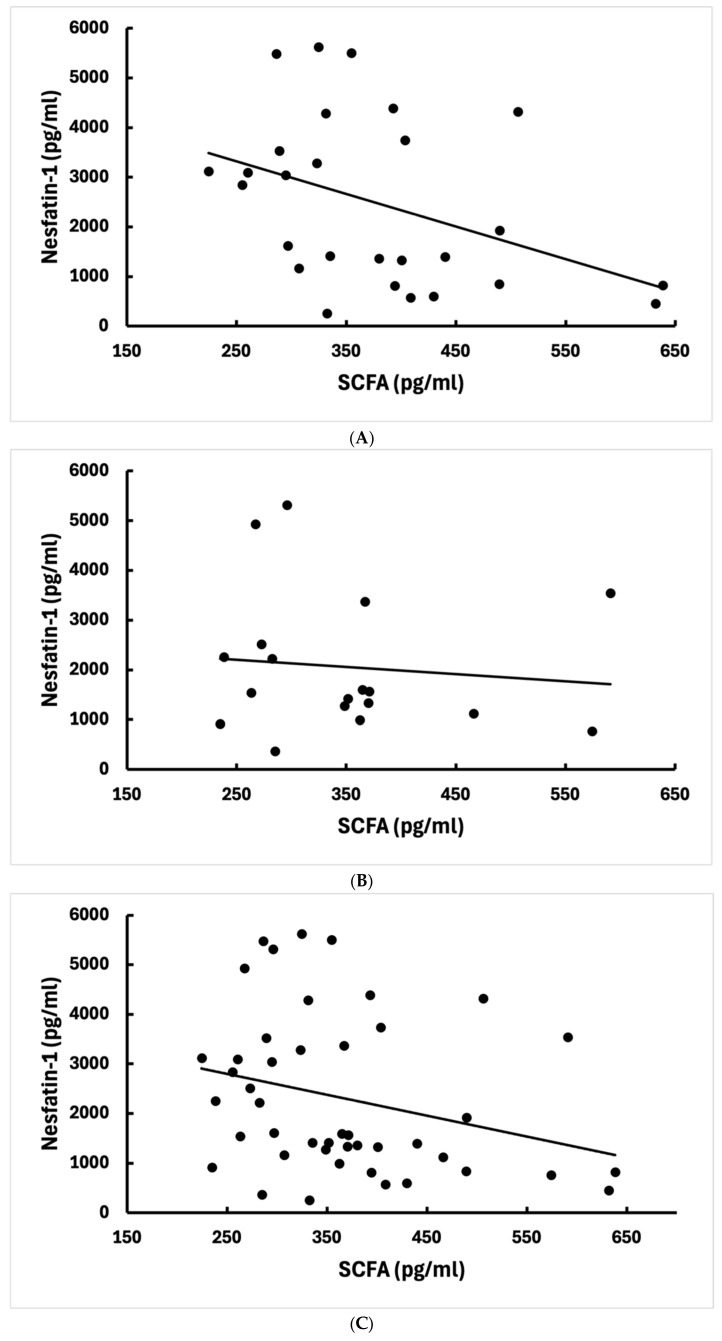
(**A**) Exploratory correlations between serum total short-chain fatty acids (SCFA) and nesfatin-1 levels in patients with ulcerative colitis (UC). Scatter plot with linear regression line. (**B**) Exploratory correlations between serum total short-chain fatty acids (SCFA) and nesfatin-1 levels in patients with Crohn’s disease (CD). Scatter plot with linear regression line. (**C**) Exploratory correlations between serum total short-chain fatty acids (SCFA) and nesfatin-1 levels in patients with IBD (CD + UC). Scatter plot with linear regression line.

We prepared box-and-whisker plots to illustrate the distribution of SCFA and nesfatin-1 levels among the three study groups (Figure 1A,B). In these plots, the central line indicates the median, the box spans the Interquartile range (IQR), and the whiskers represent the absolute range. In addition, scatter plots were generated to visualize the relationship between SCFA and nesfatin-1 levels in each group (Figure 2A–C).

Figure 1A,B: Boxplot distributions of serum total SCFA (A) and nesfatin-1 (B) levels in CD, UC, and HC. Median values and interquartile ranges are indicated.

Figure 2A–C: Scatter plots illustrating the relationship between serum total SCFA and nesfatin-1 levels in patients with IBD. Two extreme UC outliers were omitted from the plot for visualization, but were included in all statistical analyses. Panels show data for (A) patients with UC, (B) patients with CD, and (C) the combined IBD cohort. The lines represent the linear fit for visualization.

Potential outliers were evaluated using visual inspection of boxplots and scatterplots. No data points were excluded from statistical analyses; all values were retained for the computation of group comparisons and correlation coefficients, although two extreme values, one for nesfatin-1 and one for SCFAs, were omitted from graphical displays to improve readability.

Table 1 includes baseline characteristics of the study population. Table 2 summarizes the median [IQR] of SCFA and nesfatin-1 by group, Table 3 lists the Spearman correlation coefficients and *p*-values for each group and the combined IBD cohort, Table 4 lists the fecal calprotectin levels and Table 5 shows the IBDQ scores median [IQR] by group.

References were managed using the Mendeley reference management software (version 1.19.8, Elsevier, London, UK).

### 2.5. Ethical Considerations

The study protocol was approved by the hospital’s Ethics Committee and all the participants provided informed consent. The study was conducted in accordance with the Declaration of Helsinki. Participant confidentiality was maintained, and data were de-identified for analysis. There was no compensation, participation was voluntary with the option to withdraw at any time. The study posed minimal risk (a single blood draw and completing surveys).

## 3. Results

### 3.1. Baseline Characteristics of Study Population

Baseline demographic and clinical characteristics for the three study groups are shown in Table 1. A total of 78 subjects were enrolled, including 18 patients with CD, 29 patients with UC, and 31 HCs. Patients with CD and UC were comparable in terms of age, sex distribution, and smoking status. Disease duration showed wide variability in both IBD groups. Treatment profiles reflected routine clinical practice, with a substantial proportion of patients receiving biologic therapy, 5-ASA, or corticosteroids.

Among the 18 patients with CD, 9 were in remission (CDAI < 150) and 9 had active disease. Among the 29 patients with UC, 14 were in remission (Mayo ≤ 2) and 15 had active disease.

### 3.2. SCFA and Nesfatin-1 Levels in IBD vs. Controls

#### 3.2.1. SCFA Concentrations

Serum total SCFA levels did not differ significantly among the three groups (Kruskal–Wallis H = 2.46, *p* = 0.29). The median serum SCFA concentration was 350 [IQR 275–369] pg/mL in the CD group, 354 [301–434] pg/mL in the UC group, and 389 [308–444] pg/mL in healthy controls (Table 1). Although the medians differed numerically, these differences were not statistically significant.

#### 3.2.2. Nesfatin-1 Concentrations

Nesfatin-1 values differed numerically across groups, but this did not reach statistical significance (Kruskal–Wallis *p* = 0.064). Patients with CD had the lowest nesfatin-1 levels, with a median of 1553 [1118–2512] pg/mL, which was substantially lower than the median of 3077 [1915–4246] pg/mL in healthy controls. The UC group had a median nesfatin-1 of 1923 [832–4014] pg/mL, lying between CD and HC, but with a broad IQR reflecting high inter-individual heterogeneity (Figure 1B).

### 3.3. Correlation Between SCFA and Nesfatin-1

#### 3.3.1. Ulcerative Colitis

In patients with UC, we found a statistically significant inverse correlation between serum SCFA and nesfatin-1 concentrations (Spearman’s ρ = −0.47, *p* = 0.0098). As shown in Figure 2A, patients with UC with lower SCFA levels proved to have higher nesfatin-1, and vice versa.

#### 3.3.2. Crohn’s Disease

In CD, there was no significant correlation found between short-chain fatty acid and nesfatin-1 (ρ = −0.1, *p* = 0.66) (Figure 2B). The scatter of the CD data points gave the impression of randomness; several of the patients had both low levels of short-chain fatty acids and low nesfatin-1 (clusters towards the origin), while others had low levels of short-chain fatty acids but higher levels of nesfatin-1.

#### 3.3.3. Combined IBD

When looking at all patients with IBD together (CD + UC, n = 47), there was a significant negative correlation between SCFA and nesfatin-1 (ρ = −0.36, *p* = 0.012; Figure 2C).

No significant correlation was found between SCFA and nesfatin-1 in the healthy control group (ρ ≈ 0, *p* = 0.99; data not illustrated by figure).

### 3.4. Fecal Calprotectin

Fecal calprotectin values were available only for a subset of the IBD cohort and were not available for healthy controls. Values were reported in µg/g. Median calprotectin was 149 µg/g [19–770] in CD and 262 µg/g [56–764] in UC.

Exploratory descriptive analyses did not reveal consistent numerical trends between fecal calprotectin and serum SCFAs or nesfatin-1.

### 3.5. IBDQ Questionnaire Scores by Group

The median IBDQ score in patients with CD was 164 [102–197], which was lower than the median in patients with UC (190 [149–213]). The distribution suggested numerically lower scores in CD, although this difference was not statistically significant. However, there was substantial overlap in individual scores between the two groups. A Kruskal–Wallis test comparing the CD vs. UC IBDQ distributions found no statistically significant difference (H = 2.24, *p* = 0.13).

#### Correlation of IBDQ with SCFA and Nesfatin-1

Finally, we assessed whether IBDQ scores correlated with the serum biomarker levels, specifically the SCFAs and nesfatin-1 in the IBD patient cohort (n = 47). No statistically significant associations were observed between IBDQ scores and serum SCFA concentrations or nesfatin-1 levels.

## 4. Discussion

This study investigated serum short-chain fatty acids and nesfatin-1 levels in patients with IBD, revealing distinct patterns between CD and UC. To the best of our knowledge, this is one of the first analyses to specifically examine the SCFA-nesfatin-1 relationship in IBD subtypes. The central question of this study was whether circulating SCFA levels are associated with nesfatin-1 levels in patients with IBD, as a potential indicator of gut–brain axis crosstalk. We performed Spearman correlation analyses within each subgroup and in the combined IBD sample. The key finding is an inverse correlation between SCFA and nesfatin-1 in patients with UC, which was not observed in CD. This suggests that UC, but not CD, exhibits a coupling between microbial metabolic output and a host neuroendocrine response. We discuss the implications of these results in the context of existing literature on IBD, and compare them to findings in CRC, a condition that shares pathogenic links with IBD, particularly drawing on the recent study by Ilyés et al. (2025) [12], which examined SCFAs, nesfatin-1, leptin, and GLP-1 in IBD and colon cancer.

### 4.1. SCFA Levels in IBD

As per prior reports, our patients with UC showed slightly lower SCFA levels than healthy controls (median 354 vs. 389 pg/mL), although this did not reach statistical significance in our sample. The values showed wide overlap across groups (Figure 1A). Variability was considerable, indicating that most patients with IBD had lower or preserved SCFA levels. Previous studies have reported reduced serum levels of acetate and propionate in patients with IBD, supporting the concept of impaired microbial metabolic activity [18]. These findings suggest that circulating total SCFA levels alone may not clearly distinguish patients with IBD from healthy individuals, at least in a cohort of this size. Ilyés et al. report decreased SCFA levels in patients with IBD as compared to controls (exact values not given) [12]. SCFA tendency of depletion in IBD has generally been attributed to gut dysbiosis, specifically to loss of beneficial fiber-fermenting bacteria. For example, decreased abundance of butyrate-producing species like *Faecalibacterium prausnitzii* and *Roseburia* has been linked to reduced SCFA output in IBD [19,20,21]. Short-chain fatty acids (SCFAs) possess strong anti-inflammatory effects by enhancing the release of anti-inflammatory cytokines, downregulating pro-inflammatory cytokines, and blocking signaling pathways that initiate inflammatory responses. In experimental colitis, butyrate was shown to alleviate disease by reducing the expression of pro-inflammatory mediators such as interleukin-6 (IL-6), IL-17, tumor necrosis factor-alpha (TNF-α), and IL-1β [22]. Reduced SCFA availability has been associated with immune dysregulation and altered inflammatory signaling in IBD [23]. Our finding that serum SCFA levels were not dramatically different in IBD vs. controls (with considerable overlap) likely reflects the heterogeneity of disease activity and microbiota composition among patients. Measuring total SCFAs in serum may be less sensitive than fecal SCFA analysis for detecting gut microbiome changes, since serum levels are influenced by absorption and host metabolism. The trend, nevertheless, is consistent with the concept that IBD is associated with decreased SCFA-producing capacity of the microbiota [24]. It is important to note that serum SCFA measurements reflect systemic availability rather than direct luminal production. Fecal SCFAs are considered a more direct marker of gut microbial metabolic activity and dysbiosis in IBD. However, serum SCFAs may provide complementary information by capturing the fraction of microbial metabolites that are absorbed and interact with host metabolic and neuroendocrine pathways. The absence of fecal SCFA measurements in this study limits the ability to directly assess microbial production and should be considered when interpreting the findings. It is possible that analysis of individual SCFA components would reveal associations not detectable when using total SCFA concentrations.

### 4.2. Nesfatin-1 Differences in IBD

One of the key observations emerging from our analysis is the markedly lower serum nesfatin-1 levels in CD compared with HC. This is a contrast to UC, where nesfatin-1 values were more heterogeneous and not significantly different from controls as a group. This divergent pattern suggests distinct regulatory mechanisms between the two IBD subtypes.

These numerical differences may relate to nutritional status or disease-specific factors; however, confirming such associations goes beyond the scope of this study. In contrast, patients with UC displayed highly variable nesfatin-1 values, with some overlapping CD levels and others approaching or exceeding healthy ranges, suggesting that additional factors may influence nesfatin-1 in UC. The broader variability observed in UC may reflect differences in disease chronicity, extent of inflammation, or intermittent subclinical activity that activates neuroendocrine stress pathways. This dispersion suggests episodic activation of nesfatin-1 secretion related to inflammatory burden.

Anatomical localization may also be a contributing factor. CD often involves the small intestine, where nutrient absorption is most affected, whereas UC is confined to the colon. Peripheral nesfatin-1 is produced not only in adipose tissue and the hypothalamus but also in gastric and intestinal endocrine cells. Therefore, small-bowel involvement in CD could impair enteroendocrine secretion and systemic metabolic signaling, while the predominantly colonic pathology of UC might exert a different modulatory influence through local cytokine and adipokine networks.

Previous studies have reported increased nesfatin-1 levels in active IBD, with similar values in CD and UC [25]. In contrast, our study observed lower nesfatin-1 levels in CD compared to healthy controls. This discrepancy may be explained by differences in disease activity, as a substantial proportion of our CD cohort was in remission, as well as by the relatively small sample size and inter-individual variability.

The lower nesfatin-1 levels observed in CD may represent a “low nesfatin-1 phenotype” potentially associated with nutritional status or disease distribution. Future studies could be conducted in order to examine whether patients with CD with weight loss or malnutrition have lower nesfatin-1 than those without.

### 4.3. Inverse SCFA-Nesfatin Correlation in UC

One of the principal novel outcomes of our work is the finding of a modest inverse correlation between SCFA and nesfatin-1 in UC. This provides a picture of UC in which deficiency of microbial metabolites and host neurohormonal activation are interrelated. The inverse correlation observed in UC should be interpreted with caution, as it contrasts with the expected anti-inflammatory alignment between SCFAs and nesfatin-1 suggested in previous studies. Although this association reached statistical significance, it should be interpreted cautiously given the absence of correction for multiple testing.

This moderate negative correlation suggests a coupled relationship in UC. When microbial production of SCFAs is reduced, nesfatin-1 levels increase, potentially reflecting a stress or inflammatory response. Notably, this correlation remained significant even after exclusion of two potential outliers with very high nesfatin-1 values, supporting the stability of the association within the UC subgroup.

In CD, no such significant correlation was detected. This lack of association indicates that variations in SCFA availability were not linked to nesfatin-1 levels in our CD cohort. Uniformly lower nesfatin-1 levels and a limited range may have reduced the ability to detect a correlation. Differences in disease localization, particularly small bowel involvement in CD, may also contribute to distinct regulatory patterns.

The overall inverse correlation observed in the combined IBD cohort appears to be largely driven by the UC subgroup. Therefore, pooled analyses may obscure subtype-specific biomarker relationships, and these findings should be interpreted with caution.

In healthy individuals, homeostatic regulation may maintain SCFA production and nesfatin-1 within relatively independent physiological ranges, which could explain the absence of correlation in controls.

From a pathophysiological perspective, reduced colonic SCFA formation, reflecting dysbiosis in UC, may coincide with the activation of gut–brain axis pathways, potentially through immune or neural signaling. Nesfatin-1 can be produced by peripheral tissues like the colon itself (though at low levels) and by the brain in response to peripheral cytokines or vagal gut neural input. Increased nesfatin-1 could contribute to the anorexia and weight loss in active UC, effectively linking gut microbial dysfunction with central appetite regulation [26,27]. Some studies on the gut–brain axis in intestinal inflammation suggest that intestinal inflammation feeds back to the brain to alter appetite and behavior. The data that we present here, then, give a solid biomarker pair (SCFA and nesfatin-1) reference that indicates this bidirectional relationship between microbial health (i.e., availability of SCFA) and neuroendocrine response (level of nesfatin-1). One possible explanation is that nesfatin-1 may reflect a compensatory or stress-related response to inflammation or metabolic imbalance, rather than acting strictly in parallel with SCFAs.

### 4.4. Comparison with Other Contexts

Our findings and the referenced study both underscore the involvement of the gut–brain axis in IBD and IBD-associated CRC. In both scenarios, changes in gut-derived metabolites (SCFAs) correspond to changes in neuropeptides/hormones (nesfatin-1, GLP-1, leptin) that can affect appetite, metabolism, and inflammation. For instance, Ilyés et al. [12] observed that SCFAs were negatively correlated with GLP-1 in IBD and CRC, suggesting that lower SCFAs (worse dysbiosis) were linked to higher GLP-1 (possibly a compensatory response to inflammation, since GLP-1 has anti-inflammatory properties in the gut. We hypothesized that the body increases GLP-1 production in IBD as an attempt to counter inflammation, which might be insufficient naturally but is the rationale behind therapeutic GLP-1 analogs showing benefit in UC. Although glucagon-like peptide-1 was not assessed in our cohort, the inverse relationship observed between SCFAs and nesfatin-1 in UC aligns with the concept of neuroendocrine responses accompanying microbial dysregulation. Nesfatin-1, like GLP-1, could be part of an intrinsic anti-inflammatory or stress-coping response [28]. Alternatively, it might simply be a marker of inflammation severity [29,30,31]. Of interest, Beyaz et al. have demonstrated that nesfastin-1 correlated strongly with active CD with ESR (an indicator of an inflammatory process), supporting the idea that nesfatin-1 increases in inflammatory response. Also, they have reported increased nesfatin-1 levels in patients with active IBD, with similar levels in CD and UC, which contrasts with our findings [25].

### 4.5. Clinical Implications

While this was a pilot study, some possible clinical implications may be considered cautiously. Our findings indicate possible subtype-specific patterns, such as lower nesfatin-1 levels in CD and an inverse SCFA-nesfatin relationship in UC. These observations are preliminary and require validation in larger, longitudinal studies before any diagnostic or clinical utility can be inferred. For example, a UC patient with very low SCFAs and high nesfatin-1 might be identified as having active dysbiosis driven inflammation. In CD, chronically low nesfatin-1 might point to patients who have suppressed appetite signals, alerting clinicians to monitor nutritional status carefully and perhaps involve dietary interventions. Otherwise, these biomarkers are also lined in the gut–brain axis, which would indicate that therapies targeting this aspect could be the subject of additional study. It may be that restoration of the SCFA levels (by dietary or microbiota intervention) or manipulation of the nesfatin-1 signaling (though in experimental studies) could act together to ultimately control not only IBD inflammation but also symptoms such as appetite loss. SCFAs have also been implicated in extraintestinal manifestations and systemic effects of Crohn’s disease [32].

### 4.6. Limitations

Several limitations need to be acknowledged for the present study. The sample size, especially for CD, was relatively small, which affects the power to detect differences and might overemphasize the influence of a few data points. The relatively small number of patients in the CD subgroup (n = 18) may limit statistical power and the reliability of subgroup analyses, and the findings should therefore be interpreted with caution. The absence of correction for multiple comparisons increases the risk of type I error, particularly in the context of multiple correlation analyses. The cross-sectional design captures a single time-point of each patient; a longitudinal approach would be more informative to see how SCFA and nesfatin-1 co-vary with disease activity over time, as the actual study cannot establish causal relationships between the two substances with its current design. Furthermore, this was a single-center study conducted in a Romanian cohort, which may limit the external generalizability of the findings to other populations and healthcare settings. Given the limited sample size, incomplete availability of calprotectin data, and absence of healthy control measurements for calprotectin, no inferential conclusions can be drawn regarding associations between calprotectin and serum biomarkers. A key limitation of this study is that SCFAs were measured as total concentrations using ELISA-based assays, without differentiation between individual components such as acetate, propionate, and butyrate. These metabolites have distinct biological roles and may show differential associations with disease activity and immune regulation in IBD. The use of total SCFA measurements may therefore disguise specific patterns relevant to disease mechanisms. Future studies using more precise analytical techniques, such as gas chromatography-mass spectrometry, should evaluate individual SCFA fractions in order to provide a more detailed understanding of their role in IBD. The present study did not include microbiota profiling or detailed dietary assessments. Factors such as medication use, including antibiotics and probiotics, as well as disease activity, may influence SCFA and nesfatin-1 levels. SCFA levels are strongly influenced by gut microbial composition and dietary intake, particularly fiber consumption. The absence of these data limits the ability to interpret the observed variability in SCFA concentrations and to link circulating levels to specific microbial or nutritional patterns.

### 4.7. Further Directions

Further research should validate the inverse SCFA-nesfatin correlation in a larger UC cohort and determine if it holds true in active disease states. It would also be interesting to explore the mechanisms involved, using either in vitro or in vivo models, which could help clarify whether alterations in SCFA availability, for example due to dysbiosis, influence nesfatin-1 expression through inflammatory pathways. Experiments can be done to find if replacement of SCFAs or restoring the microbiota can reduce nesfatin-1 and improve anorexia/inflammation in models of colitis. Furthermore, as interest increases in the gut–brain axis, evaluation of symptoms such as appetite, nausea, pain, mood, of the patients in correlation with SCFA and nesfatin-1 measures could provide insights into how these biomarkers correlate with clinical expressions of IBD.

Our study suggests a potential dual-pattern, namely lower nesfatin-1 levels in CD and an inverse SCFA-nesfatin association in UC. These findings are hypothesis-generating and should be confirmed in larger, well-phenotyped cohorts. By comparing our IBD findings with those in colorectal cancer and prior IBD studies, we observe common threads, namely, that dysbiosis and inflammation are linked with changes in appetite-regulating hormones across these conditions. Our results support the concept that an abnormal gut microbiome (as seen in SCFA-depleted states) can have a systemic neuroendocrine effect (i.e., increased nesfatin-1) which may ultimately contribute to disease symptoms and outcomes.

SCFAs are important microbial metabolites that regulate gut health, as well as produce an anti-inflammatory effect in IBD [33,34]. Reduced SCFA availability has been associated with impaired epithelial barrier function and altered immune responses in IBD, and SCFAs are recognized mediators of microbiota–gut–brain axis signaling [27,35,36]. Future studies should integrate microbiota profiling and standardized dietary assessments to better characterize the relationship between microbial metabolism, circulating SCFAs, and host neuroendocrine responses in IBD and should incorporate multivariable or stratified analyses to account for potential confounders such as diet, medication use, and disease activity.

Nesfatin-1 is now considered one of the key “gastrokines” involved in gut–brain axis signaling, alongside hormones like ghrelin [37]. Its role in IBD remains as of yet partially unknown, and it is unclear whether changes in circulating nesfatin-1 reflect pro-inflammatory activity or compensatory responses to inflammation [5,38]. Further studies are required to clarify its biological significance in chronic intestinal inflammation.

Quality of life was impaired in both CD and UC, with no significant difference between subtypes. No statistically significant associations were demonstrated between serum SCFA concentrations or nesfatin-1 levels and IBDQ scores. Overall health-related quality of life impairment appeared comparable between patients with CD and UC in this cohort. Both subtypes demonstrated substantial quality-of-life burden. The combined IBD median score of 183 fell between subtype medians, reflecting aggregate disease impact across the cohort. Consequently, the present data do not support a relationship between these circulating biomarkers and patient-reported quality of life. Any numerical tendencies observed should be considered exploratory only and interpreted with caution, particularly given the limited sample size and cross-sectional design. Longitudinal studies are needed to clarify the temporal and causal relationships between SCFAs, nesfatin-1, and disease activity in IBD.

## 5. Conclusions

In adults diagnosed with inflammatory bowel disease, distinct, exploratory differences were observed between UC and CD in analysis of serum SCFA and nesfatin-1. Patients with CD, particularly those in remission, showed an independent low-nesfatin-1 profile despite normal SCFA levels, suggesting a unique metabolic or neuroendocrine phenotype. In contrast, ulcerative colitis patients demonstrated a clear inverse correlation between SCFA and nesfatin-1 levels, and lower microbial SCFA output was associated with higher nesfatin-1, underscoring a link between gut dysbiosis and host neurohormonal stress signaling in UC.

Comparing our results to related observations in colon cancer and IBD, we note that similar biomarker behaviors occur in these contexts: both severe inflammation and neoplasia can drive up nesfatin-1 and disrupt SCFA dynamics. However, unlike colorectal cancer, where nesfatin-1 was universally high and correlated with SCFA depletion, IBD shows a spectrum dependent on disease type and activity, highlighting the complexity of the gut–brain interplay. Our findings highlight that examining microbial metabolites alongside neuropeptides provides valuable insights into disease mechanisms. Targeting the gut-microbiota-brain axis, for instance by restoring SCFA-producing microbiota or modulating anorexigenic signaling, may emerge as a complementary strategy in managing IBD. Taken together, these findings are hypothesis-generating. This pilot exploratory study suggests a potential subtype-specific association, particularly in UC; however, given the limited sample size, especially in the CD group, these findings should be interpreted with caution. Larger, well-powered studies are required to confirm these observations.

## Figures and Tables

**Table 1:** Baseline demographic and clinical characteristics of patients with CD, UC, and HC included in the study.

Characteristic	CD (n = 18)	UC (n = 29)	Healthy (n = 31)
**Age, years (mean ± SD)**	45 ± 16.6	47 ± 16.6	44 ± 17.5
**Female, n (%)**	11 (61.1%)	14 (48.3%)	16 (51.6%)
**BMI, kg/m^2^, Median [IQR]**	22.7 [21.5–25.4]	24.3 [21.6–28.9]	25.15 [23–27.6]
**Smoking status, n (%)**			
Never	9 (50%)	13 (44.8%)	14 (45.2%)
Former	6 (33.3%)	10 (34.5%)	11 (35.5%)
Current	3 (16.7%)	6 (20.7%)	6 (19.4%)
**Disease duration, years, Median [IQR]**	7 [5–12]	7 [2–17.5]	-
**Biologic therapy, n (%)**			-
Anti-TNF	8 (44%)	10 (34.5%)	-
Anti-Integrin	3 (16.7%)	8 (27.6%)	-
JAK Inhibitor	0	1 (3.4%)	-
**AZA use, n (%)**	2 (11.1%)	5 (17.2%)	-
**5-ASA use, n (%)**	0	19 (65.5%)	-
**Corticosteroids, n (%)**	2 (11.1%)	5 (17.2%)	-

SD = Standard Deviation, BMI = Body Mass Index, IQR = Interquartile range, Anti-TNF = Anti-Tumor Necrosis Factor, JAK = Janus Kinase, AZA = Azathioprine, 5-ASA = 5-aminosalicylic acid.

**Table 2:** Serum total short-chain fatty acid and nesfatin-1 concentrations in patients with Crohn’s disease (CD), ulcerative colitis (UC), and healthy controls (HC).

Group	SCFA (pg/mL), Median [IQR]	Nesfatin-1 (pg/mL), Median [IQR]
CD (n = 18)	350 [275–369]	1553 [1118–2512]
UC (n = 29)	354 [301–434]	1923 [832–4014]
HC (n = 31)	389 [308–444]	3077 [1915–4246]
Kruskal–Wallis *p*	0.29	0.064

Values are expressed as median [IQR]. *p*-values were obtained using the Kruskal–Wallis test. Abbreviations: SCFA, short-chain fatty acids; IQR, interquartile range; CD, Crohn’s disease; UC, ulcerative colitis; HC, healthy controls.

**Table 3:** Spearman rank correlation analysis between serum total short-chain fatty acid (SCFA) concentrations and nesfatin-1 levels in patients with Crohn’s disease (CD), ulcerative colitis (UC), the combined IBD cohort, and healthy controls (HC). Shown are Spearman’s rank correlation coefficients (ρ) and *p*-values.

	Spearman ρ (SCFA vs. Nesfatin-1)	*p*-Value
**CD (n = 18)**	−0.1	0.66
**UC (n = 29)**	−0.47	0.0098
**Combined IBD (n = 47)**	−0.36	0.012
**Healthy (n = 31)**	−0.15	0.39

*p* < 0.05 (significant); *p* < 0.01 (highly significant). Abbreviations: SCFA, short-chain fatty acids; CD, Crohn’s disease; UC, ulcerative colitis; IBD, inflammatory bowel disease; HC, healthy controls; ρ, Spearman’s rank correlation coefficient.

**Table 4:** Exploratory descriptive fecal calprotectin concentrations and their associations with serum total SCFAs and nesfatin-1 in patients with Crohn’s disease (CD), ulcerative colitis (UC) and the combined Inflammatory Bowel Disease (IBD) cohort.

Group	Calprotectin (µg/g) Median [IQR]	Exploratory Association with Nesfatin-1 (ρ, *p*)	Exploratory Association with SCFA (ρ, *p*)
**CD (n = 9)**	149 [19–770]	0.30, *p* = 0.43	−0.10, *p* = 0.79
**UC (n = 17)**	262 [56–764]	−0.10, *p* = 0.68	−0.14, *p* = 0.58
**Combined IBD (n = 26)**	204 [49–739]	0.07, *p* = 0.73	−0.04, *p* = 0.83

*p* < 0.05 (significant); *p* < 0.01 (highly significant). ρ indicates Spearman’s rank correlation coefficient. Abbreviations: CD, Crohn’s disease; UC, ulcerative colitis; IBD, inflammatory bowel disease; SCFA, short-chain fatty acids; IQR, interquartile range. Note: Fecal calprotectin data were available only for a subset of patients with IBD and were not available for healthy controls. Reported associations are descriptive and exploratory only, and no inferential conclusions should be drawn.

**Table 5:** Inflammatory Bowel Disease Questionnaire (IBDQ) total scores (median and IQR) in patients with Crohn’s disease (CD), ulcerative colitis (UC) and the combined Inflammatory Bowel Disease (IBD) cohort.

Group	N	IBDQ Median (IQR)
**CD**	18	164 (102–197)
**UC**	29	190 (149–213)
**Combined IBD**	47	183 (137–211)

Abbreviations: IBDQ, Inflammatory Bowel Disease Questionnaire; IBD, inflammatory bowel disease; CD, Crohn’s disease; UC, ulcerative colitis; IQR, interquartile range. Note: Higher IBDQ scores indicate better disease-specific quality of life. No significant difference was observed between CD and UC.

## Data Availability

The dataset used in this study is part of an ongoing research program and is therefore not publicly available. Anonymized data can be provided by the corresponding author upon reasonable request, in accordance with institutional and ethical regulations.

## Abbreviations

The following abbreviations are used in this manuscript:

SCFAs	Short-chain fatty acids
IBD	Inflammatory bowel disease
CD	Crohn’s disease
UC	Ulcerative colitis
HC	Healthy controls
QoL	Quality of life
ELISA	Enzyme-linked immunosorbent assay
IBDQ	Inflammatory Bowel Disease Questionnaire
ECCO	European Crohn’s and Colitis Organisation
IQR	Interquartile range
ρ	Spearman’s rho (correlation coefficient)
SD	Standard Deviation
BMI	Body Mass Index
Anti-TNF	Anti-Tumor Necrosis Factor
JAK	Janus Kinase
K–W	Kruskal–Wallis (test)
NUCB2	Nucleobindin-2
GLP-1	Glucagon-like peptide-1
FFAR2 (GPR43)	Free fatty acid receptor 2 (G protein–coupled receptor 43)
FFAR3 (GPR41)	Free fatty acid receptor 3 (G protein–coupled receptor 41)
CRC	Colorectal cancer
STROBE	Strengthening the Reporting of Observational Studies in Epidemiology
ESR	Erythrocyte sedimentation rate
AZA	Azathioprine
5-ASA	5-aminosalicylic acid
TNF-α	Tumor necrosis factor alpha
IL-6	Interleukin-6
IL-17	Interleukin-17
IL-1β	Interleukin-1 beta
